# Obsessive–compulsive disorder induced by donepezil in a patient with Alzheimer's disease

**DOI:** 10.1002/pcn5.217

**Published:** 2024-06-17

**Authors:** Kohei Echizen, Daisuke Hirose

**Affiliations:** ^1^ Department of Psychiatry Toranomon Hospital Tokyo Japan; ^2^ Department of Geriatric Medicine Toranomon Hospital Tokyo Japan

**Keywords:** Alzheimer's disease, donepezil, obsessive–compulsive disorder, stroke

## Abstract

**Background:**

Donepezil, an acetylcholinesterase inhibitor commonly used to treat Alzheimer's disease (AD), is generally well tolerated. There have been no previous reports on donepezil‐induced obsessive–compulsive disorder (OCD).

**Case Presentation:**

The patient, a retired man in his 70s diagnosed with AD, displayed OCD symptoms following donepezil initiation, exacerbating post‐stroke—specifically, a cerebral infarction in the right posterior limb of the internal capsule. Remarkably, the symptoms abated upon discontinuation of donepezil.

**Conclusion:**

Caution should be exercised when using donepezil in patients with a history of stroke.

## Graphical abstract

The patient, a retired man in his 70s diagnosed with Alzheimer's disease, displayed obsessive–compulsive symptoms following donepezil initiation, exacerbating post‐stroke—specifically, a cerebral infarction in the right posterior leg of the internal capsule. Remarkably, the symptoms abated upon discontinuation of donepezil.

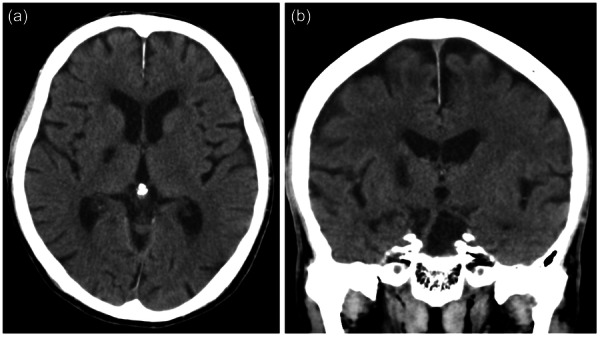

## BACKGROUND

Obsessive–compulsive disorder (OCD) is presumed to involve both serotonergic and dopaminergic systems.^1^ Donepezil is an acetylcholinesterase inhibitor commonly used to treat Alzheimer's disease (AD). Its adverse effects include nausea and anorexia as well as neuropsychiatric symptoms, such as agitation and somnolence.^2^ This case report presents a unique instance of donepezil‐induced OCD in a patient with AD.

## CASE PRESENTATION

**Figure 1:**
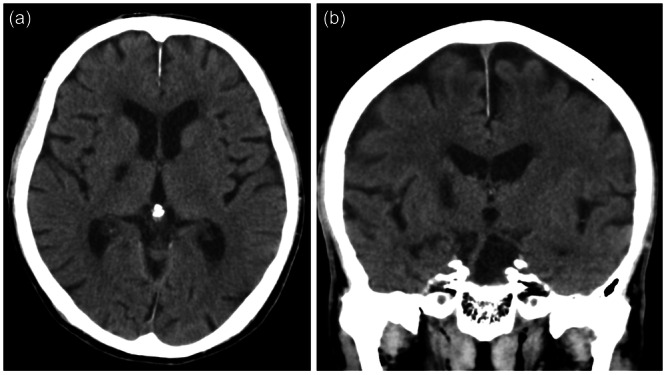
(a) Axial and (b) coronal computed tomography images taken 1 month after infarction.

The patient was a retired man in his 70s. He had no medical or mental illness and was independent in activities of daily living. The patient had no family history of dementia. In year X−2, he became prone to losing things and getting dates mixed up. In June X−1, the patient was referred to a geriatrician for memory impairment. Mini‐Mental State Examination revealed disorientation and short‐term memory impairment, with a score of 25. The Japanese version of the Montreal Cognitive Assessment (MoCA‐J) score was 22 points, with losses mainly due to delayed reproduction. The Global Clinical Dementia Rating score was 0.5, suggesting mild cognitive impairment (MCI). Magnetic resonance imaging revealed no major cerebrovascular disease; however, atrophy was limited to the hippocampal region. ^123^I‐iodoamphetamine cerebral perfusion single‐photon emission computed tomography (SPECT) revealed decreased regional cerebral blood flow (rCBF) in the left parietal lobe, bilateral precuneus, and bilateral posterior cingulate gyrus. Although decreased accumulation was also observed in the occipital lobes, there were no symptoms suggestive of dementia with Lewy bodies (DLB). The patient was diagnosed with MCI due to AD. His amnesia gradually worsened, and on June 4, X, the MoCA‐J score decreased to 21 points. SPECT showed worsening of the previously observed decrease in rCBF. Donepezil was initiated at 3 mg and increased to 5 mg 2 weeks later for AD dementia. After increasing the dose, he became worried that children might fall into the irrigation canal at his parents' house in another prefecture and that the field he leased to another person might expand beyond its original boundaries. On July 26, he was hospitalized because of a cerebral infarction around the right posterior limb of the internal capsule (Figure 1). Clopidogrel and atorvastatin were administered. Dysarthria and left hemiparesis occurred temporarily; however, no physical residual impairments remained. Around August 10, he became increasingly anxious and repeatedly and intrusively conjured up images of solar panels being installed on the field without his permission, though no such thing was happening. There were no hallucinations or other symptoms suggestive of delirium or DLB. Symptoms persisted even after discharge on August 14. On September 10, he visited the field and confirmed the absence of solar panels. Even after that, despite remembering that he had checked the site, the image still came to his mind and his anxiety persisted. On September 15, he consulted a psychiatrist on referral from the geriatrician. He stated “I remember that I visited the field. I remember there were no solar panels. But the vision of the solar panels does not go away. It is painful to visualize things that are not there,” indicating that he recognized that the obsessive imagery was not real. He said “When I start worrying about it, that is all I can think about. I am unable to sleep at night.” The patient had only obsessive thoughts and no compulsive behaviors. There were no other obsessions or compulsions, such as hand washing or door checking. Concerns were limited to the above issues and not generalized. Under the diagnosis of OCD possibly due to donepezil administration, the drug was discontinued. At the next visit a month later, his concerns had disappeared. His wife said that he was calm, as usual.

## DISCUSSION

The symptoms of conjuring up images of solar panels can be classified as obsessive thoughts, not delusions, because the patient recognized that they were not true. The obsessive thoughts caused him distress. Since he retained the memory of confirming that the solar panels were not there, it can be concluded that his repeated worrying was not attributed to cognitive decline.

The patient had no history of OCD. The obsessions that appeared after donepezil administration worsened after stroke and resolved after donepezil cessation. This suggests that OCD symptoms were caused by donepezil. There have been no previous reports on donepezil‐induced OCD. Although one report showed improvement in OCD with donepezil, it was a small case series with no blinding or placebo control.^3^

The mechanism by which donepezil causes OCD remains unclear. Cerebral infarction may have played a role. Donepezil can cause agitation or depression as adverse effects.^4^ Depression and OCD often co‐occur and may partially share etiologies.^5^ The patient's stroke involved the corticostriatal–thalamic–cortical loop, whose injury has been shown to cause or ameliorate OCD.^6^, ^7^ It is possible that the stroke made the patient prone to OCD and that the adverse events appeared as OCD instead of agitation or depression. In other words, the “two hits” of infarction and donepezil may have induced OCD.

This case suggests a cholinergic involvement in the pathogenesis of OCD. Several reports have shown that cholinergic disturbances are involved in OCD; acetylcholine release in the striatum is elevated in OCD mouse models.^8^ Magnetic resonance spectroscopy has revealed elevated choline levels in the striata of some patients with OCD.^9^, ^10^ Meanwhile, the striatum is shown to be hypoactive in patients with pediatric autoimmune neuropsychiatric disorders associated with streptococcal infections, which causes OCD symptoms in children, due to antibodies specifically binding to the cholinergic interneuron.^11^ Because there is no consensus on the role of the cholinergic system in OCD, further studies are needed.

## CONCLUSION

The present case suggests that donepezil can cause OCD symptoms. Caution should be exercised when using donepezil in patients with a history of stroke.

## AUTHOR CONTRIBUTIONS

Kohei Echizen and Daisuke Hirose treated the patient. Kohei Echizen wrote the first draft and Daisuke Hirose revised it.

## CONFLICT OF INTEREST STATEMENT

The authors declare no conflict of interest.

## ETHICS APPROVAL STATEMENT

This study was conducted according to the principles of the Declaration of Helsinki.

## PATIENT CONSENT STATEMENT

Written informed consent for the publication of this report was given by the patient.

## CLINICAL TRIAL REGISTRATION

N/A

## Data Availability

N/A
